# Spirolides in Bivalve Mollusk of the Galician (NW Spain) Coast: Interspecific, Spatial, Temporal Variation and Presence of an Isomer of 13-Desmethyl Spirolide C

**DOI:** 10.3390/toxins15010013

**Published:** 2022-12-24

**Authors:** Juan Blanco, Fabiola Arévalo, Ángeles Moroño, Jorge Correa, Araceli E. Rossignoli, Juan Pablo Lamas

**Affiliations:** 1 Centro de Investigacións Mariñas, Xunta de Galicia, Pedras de Corón, 36620 Vilanova de Arousa, Spain; 2 Instituto Tecnolóxico para o Control de Medio Mariño de Galicia (INTECMAR), Xunta de Galicia, Peirao de Vilaxoán s/n, 36611 Vilagarcía de Arousa, Spain

**Keywords:** spirolides, cyclic imines, isomer, mollusk, bivalves, monitoring, sentinel

## Abstract

Spirolides are cyclic imines whose risks to human health have not been sufficiently evaluated. To determine the possible impact of these compounds in Galicia (NW Spain), their presence and concentration in bivalve mollusk were studied from 2014 to 2021. Only 13-desmethyl spirolide C (13desmSPXC) and an isomer have been detected, and always at low concentrations. Mussel, *Mytilus galloprovincialis,* was the species which accumulated more spirolides, but the presence of its isomer was nearly restricted to cockle, *Cerastoderma edule*, and two clam species, *Venerupis corrugata* and *Polititapes rhomboides*. On average, the highest 13desmSPXC levels were found in autumn-winter, while those of its isomer were recorded in spring-summer. Both compounds showed decreasing trends during the study period. Geographically, the concentration tends to decrease from the southern to the north-eastern locations, but temporal variability predominates over spatial variability.

## 1. Introduction

**Figure 1:**
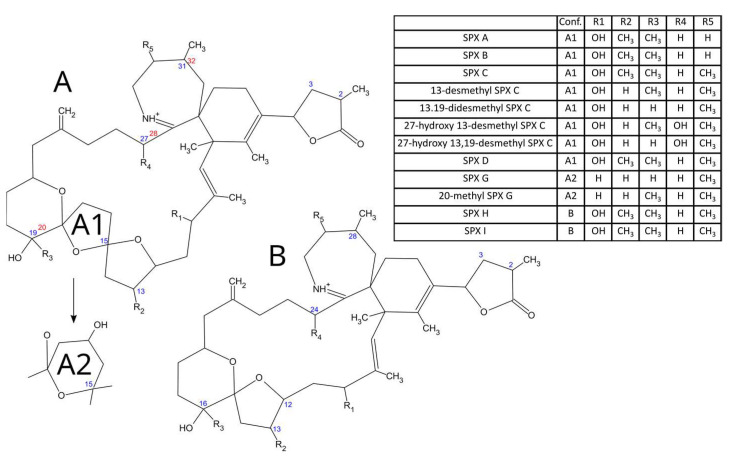
Structures of the main spirolides. A1, A2 and B are the molecular conformations which appear in the column Conf. of the table.

Spirolides are compounds characterized by a cyclic imine ring in a macrocycle system that also contains a trispiroketal ring system (Figure 1). Currently, more than 15 analogs have been structurally identified [1]. They were first identified in methanolic extracts of the digestive glands of mussels and scallops from Nova Scotia (Canada) by Hu et al. [2], but they are known to be distributed worldwide and have been reported in Scotland [3], Norway [4], the USA [5], Italy [6], Spain [7], Denmark [8], France [9], the Netherlands [10], Ireland [11], Croatia [12], Chile [13], Mexico [14], China [15], and Argentina [16,17]. Spirolides are “fast-acting toxins” that kill mice in a very short time when administered intraperitoneally [18,19]. However, their oral toxicity is much lower [20]. No human intoxication by these group of compounds has been reported [10], but some vague symptoms (such as gastric distress or tachycardia) have been reported during the months in which these toxins were frequently found in shellfish of Nova Scotia, Canada [21]. Spirolides and other cyclic imines have been shown to interact with nicotinic and/or muscarinic acetylcholine receptors, thereby affecting the muscular and nervous systems of mammals [22,23]. Therefore, there is some concern regarding the possible impact of these compounds on human health. Notwithstanding, the scarcity of data regarding spirolide occurrence to evaluate the actual risk has led the European Food Safety Authority to ask for an increase in the number of analyses of these toxins in commercial shellfish species [10].

Spirolides were first found to be produced by the dinoflagellate *Alexandrium ostenfeldii* in Nova Scotia, Canada [24]. To date, they have not been linked to any other species (considering *A. peruvianum* to be a synonym of *A. ostenfeldii*). The toxin profiles of *A. ostenfeldii* strains from different areas have been shown to be different [8,25,26,27,28,29,30].

In a recent study carried out on European commercial bivalves, 13-desmethyl spirolide C (13desmSPXC) was found in 16.7% of the analyzed samples, and 9.4% were identified with concentrations above 25 µg kg^−1^, but no relevant information about the temporal and spatial variability was obtained [31]. In Galicia, the presence of spirolides was first reported in bivalves in 2006 [7] and subsequently, in bivalves, water columns, and sediments (mostly in dinoflagellate cysts) [32,33], but always at low concentrations. Only 13desSPXC was detected. Since 2013, 13desSPXC and other cyclic imines have been included in the routine monitoring of Galician bivalve production areas carried out by INTECMAR.

**Figure 2:**
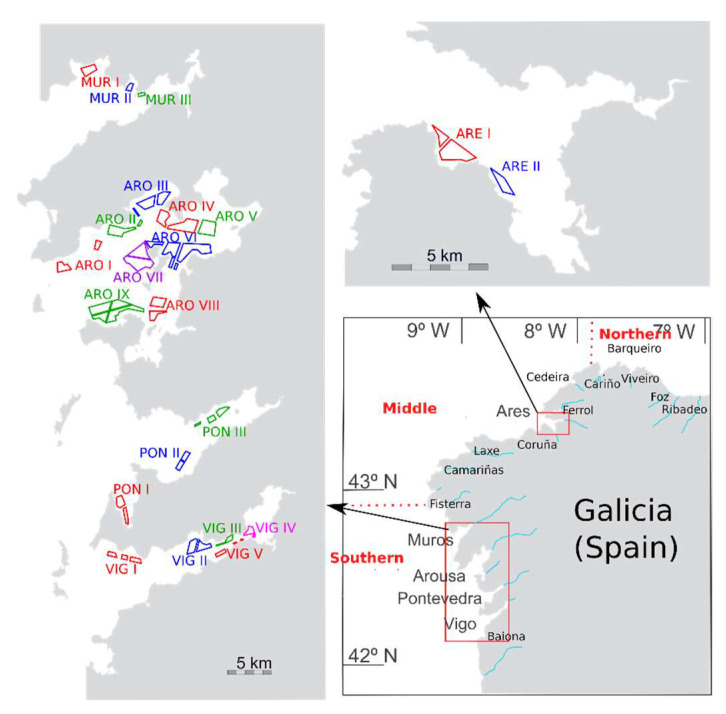
Area of study (Galicia, NW Spain). Rías from which samples were collected (lower right panel), as well as mussel production areas (two other panels), from 2014 to 2021.

In this study, spirolide data gathered by INTECMAR were analyzed to determine the toxin profiles, as well as the interspecific, temporal, and spatial variability of 13desmSPXC in the area (Figure 2).

## 2. Results

### 2.1. Toxin Profile

**Figure 3:**
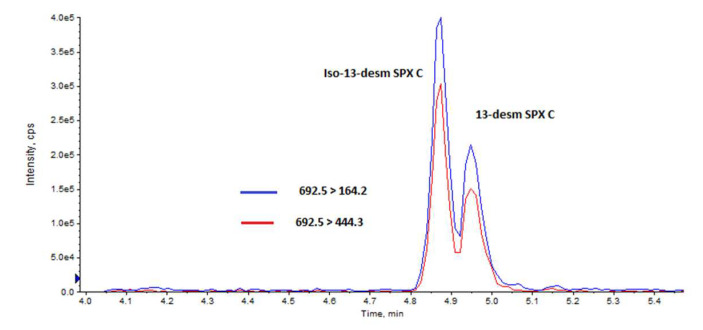
Chromatogram showing the peaks corresponding to 13-desmethyl spirolide C and its isomer for the two fragments routinely used to monitor 13-desmethyl spirolide C.

**Figure 4:**
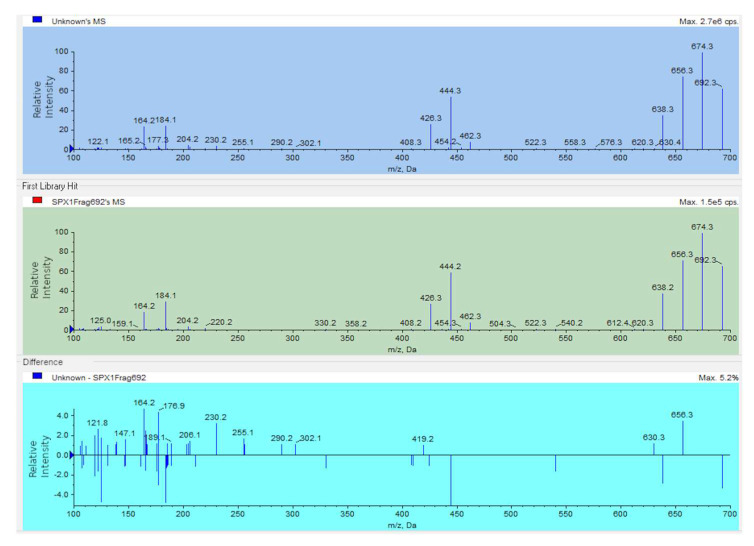
MS2 fragmentation spectra of 13-desmethyl spirolide C (top panel), its isomer (middle panel), and differences between them (lower panel).

After searching for 15 spirolide analogs, only 13desmSPXC and an isomer (Iso-13desmSPXC) were found in the analyzed samples (Tables S1–S3). Iso-13desmSPXC elutes earlier than 13desmSPXC in the chromatogram (Figure 3) and has practically the same MS2 (Figure 4) and MS3 (Figures S1 and S2) fragmentation patterns, consequently showing good agreement when compared to the library stored spectra of 13desmSPXC (Table 1).

**Figure 5:**
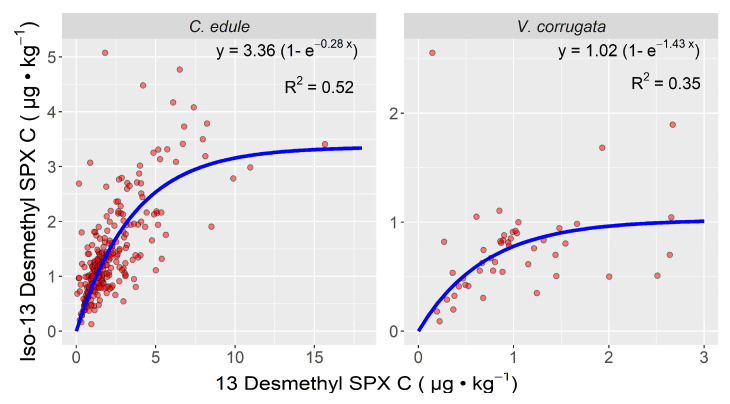
Relationship between the concentration of 13-desmethyl spirolide C and its isomer in the two bivalve species in which the isomer was detected in more than 10 samples.

In the two bivalve mollusk species in which the isomer was detected in more than 10 samples (the cockle *Cerastoderma edule* and the clam *Venerupis corrugata*), the ratio between the two compounds decreased with increasing concentrations of 13desmSPXC. In the cockle, the ratio was much higher than in the clam *V. corrugata* (Figure 5.)

### 2.2. Inter-Specific Variability

**Figure 6:**
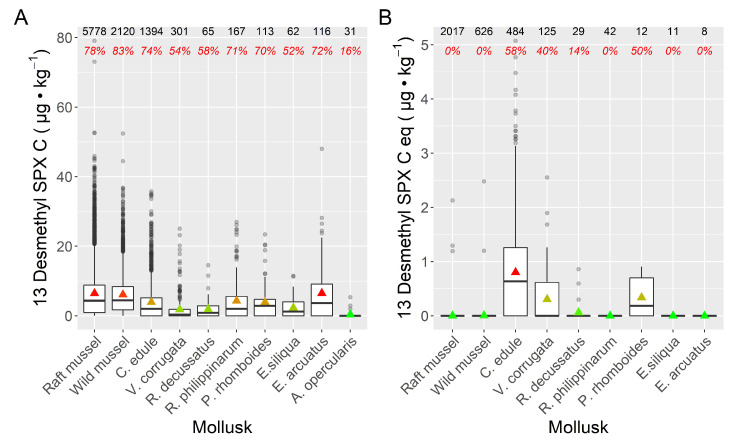
Concentration of 13 desmethyl spirolide C (**A**) and its isomer (**B**) in different bivalve species. Raft and wild mussels are *Mytilus galloprovincialis*. Triangles = means (with color gradient from red to green corresponding to maximum to minimum); horizontal lines in the box = 25, 50, and 75% quantiles; extreme vertical lines issuing from the box = range excluding outliers; dots = outliers. The numbers at the top are the number of samples analyzed and the percentages of samples in which the compounds were detected (prevalence).

Mussels, *Mytilus galloprovincialis,* showed the highest prevalence of 13desmSPXC, followed by the razor clam *Ensis arctuatus*, the cockle *C. edule*, and the clams *Ruditapes philippinarum* and *Polititapes rhomboides*. Two other clams, *V. corrugata* and *Ruditapes decussatus*, as well as the razor clam, *Ensis siliqua*, and the pectinid, *Aequipecten opercularis*, had the lowest 13desmSPXC prevalence. The average toxin levels followed the same trend as the prevalence (Figure 6).

The isomer exhibited a very different pattern. The maximum prevalence and average concentrations were observed in the cockle *C. edule*, and in some clams (*V. corrugata*, *P. rhomboides* and *R. decussatus*), to a much lower extent. Zero or nearly zero prevalence and average concentrations were recorded in all other species, including mussels (both wild and raft-cultured populations) (Figure 6).

More than 75% of the obtained 13desmSPXC results were below 10 µg kg^−1^. The maximum concentration attained was detected in raft mussels, and was slightly below 80 µg kg^−1^ (Figure 6A). In general, the concentrations of 13desmSPXC were substantially higher than those obtained for its isomer (Figure 6). However, on average, when the isomer was detected in *C. edule* and *V. corrugate*, its levels were close to those of 13desmSPXC (Figure 6B), and the proportions of this compound relative to 13desmSPXC were 1.15 and 0. 95, respectively. In no case was the guide level proposed by the EU Reference Laboratory for Marine Biotoxins, CRLMB (400 µg kg^−1^), attained [10].

In general, there was a positive correlation of 13desmSPX levels among the studied bivalves (Figures S3 and S4). The exceptions to this positive correlation with mussels, and between them, respectively, were those of *A. opercularis* and *R. decussatus*, which were species with a low number of samples analyzed.

### 2.3. Spatial Variability

**Figure 7:**
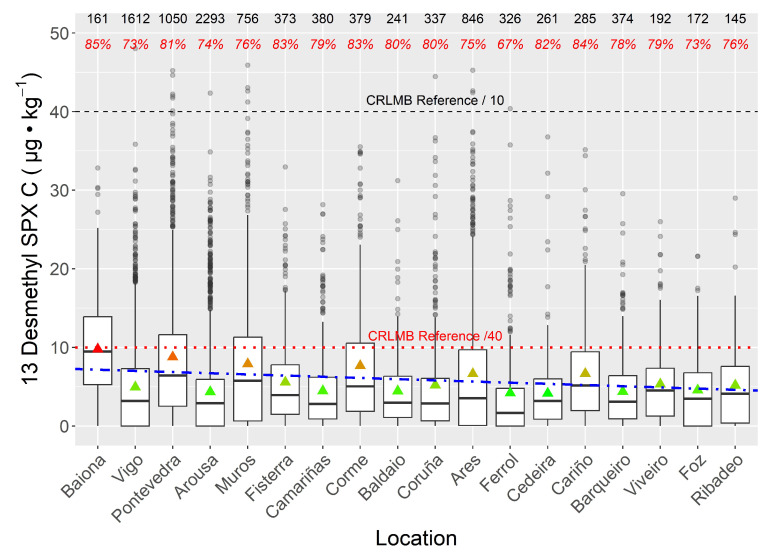
The 13-desmethyl spirolide C concentrations in bivalves from the studied locations. The numbers at the top are the number of samples analyzed and the percentages of samples in which the compounds were detected (prevalence). The blue dashed-dotted line shows the south-northeast trend. All other aspects are the same as those in Figure 6.

When the data for all species were combined, no large differences were found among locations. A slight decreasing trend in spirolide concentrations from south (Baiona) to northeast (Ribadeo) appears to be present, but the relatively high concentrations found in Corme, Ares, Ferrol, and Cariño indicate the existence of a local component (Figure 7). 

When only wild mussels (which were not sampled in all areas) were included in the analysis, the general trend was the same (Figure S5), but the recorded levels in the Ría of Coruña were higher than expected. For cockles, when locations with less than three observation were excluded, the decreasing trend persists (Figure S6), but with a much lower slope than when mussels or all bivalve species were included in the analysis. 

**Figure 8:**
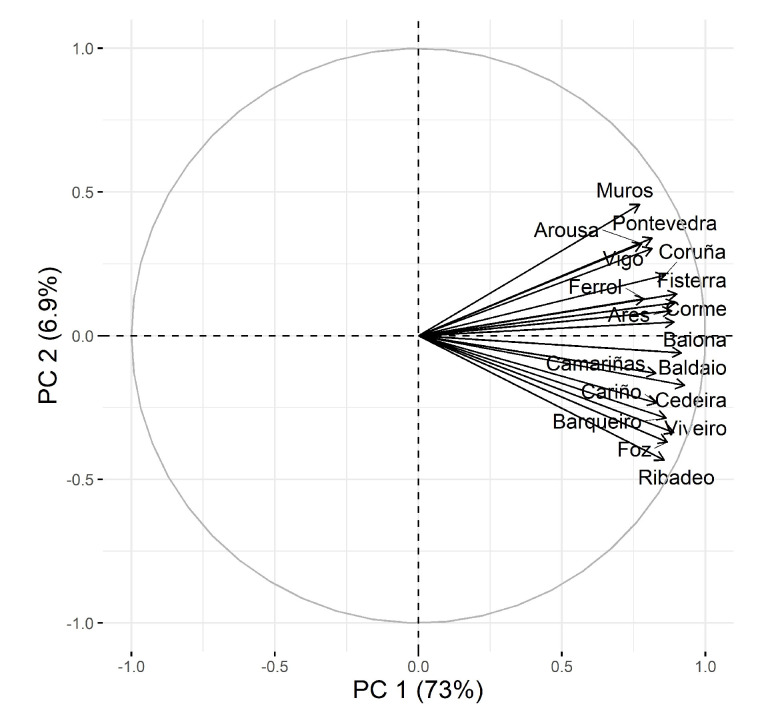
Principal component analysis of 13-desmethyl spirolide SPXC concentrations in bivalves at the sampling locations of Galicia.

Principal component analysis (PCA) extracted a first component, which explains more than 72% of the variance and was related to all locations together (Figure 8), indicating that most of the variation in toxin concentration occurred simultaneously in all areas, and that these variations are not dependent on location, but are basically temporal variations. The second component explains a more reduced proportion of the variation (7%) and seems to be linked to the south-northeast gradient in the sampling locations.

### 2.4. Temporal Variability

#### 2.4.1. Seasonality

**Figure 9:**
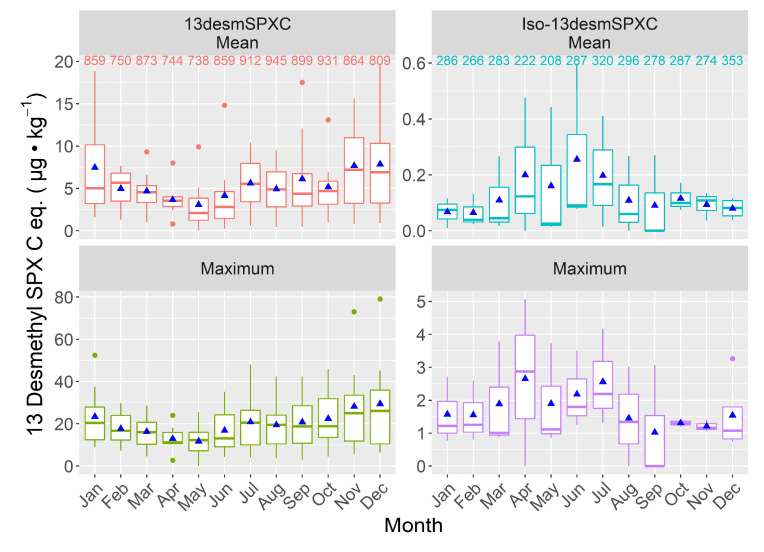
Seasonal variation in the mean and maximum levels of 13-desmethyl spirolide C and its isomer during the study period. The box description and other symbols are the same as those in Figure 6.

The highest values of both the mean and maximum levels attained for 13desmSPXC occurred in late autumn-early winter, and the lowest at the end of summer. The isomer, however, showed its maximum levels in spring-summer and minimum levels in late-autumn-winter (Figure 9). This difference is maintained when only the samples for which both compounds were analyzed are used (Figure S7).

The temporal variability is higher than the geographical variability, as could also be expected from the high PC1 loading (Table S4).

#### 2.4.2. Interannual Variation

**Figure 10:**
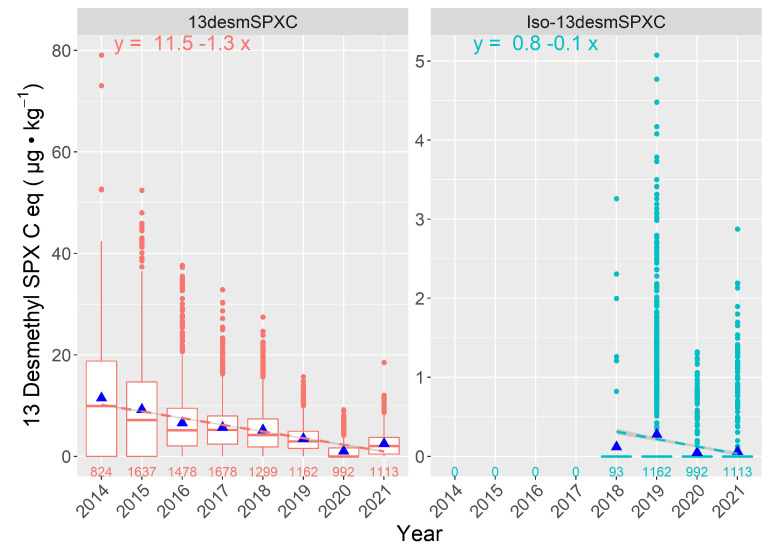
The 13-desmethyl spirolide C and Iso-13-desmethyl spirolide C concentration in bivalves during the study period (13-desmethyl spirolide C has only been analyzed since 2018). The box description are the same as those in Figure 6. Numbers at the bottom are the samples analyzed.

The measured 13desmSPXC levels progressively decreased from 2014 to 2020, with a slight increase in 2021 (Figure 10). The trend of Iso-13desmSPXC concentration is less clear, but only data from 2018 to 2021 are available.

## 3. Discussion

To date, only two spirolides have been detected in mollusks from Galicia, 13desmSPXC and an isomer that is described in this study. The isomer has a fragmentation profile very similar to that of 13desmSPXC and has been shown to be produced by enzymatic transformation in some mollusks [34]. The fragmentation spectra of the isomer, obtained using double (MS2) and triple-stage mass spectrometry (MS3), showed fragmentation products that were very similar to those of 13desmSPXC. This suggests that the isomer may be an epimer of 13desmSPXC. Epimers of some cyclic imines, such as pinnatoxins B/C [35,36] and gymnodimine B/C [37], have been documented in bivalves; however, hitherto they have not been linked to transformations in invertebrates. 

The production of 13desmSPXC has been observed in several strains of *Alexandrium ostenfeldii* [8,16,25,29,30,38,39,40,41], and its presence in bivalves or *A. ostenfeldii* cultures from different areas, including Galicia, has been reported [7,9,11,13,14,17,26,28,31,37,38,41,42,43,44]. Other spirolides detected on the Atlantic coast of Europe, include spirolide A, 13-desmethyl spirolide D [9,39,45], 20-methyl spirolide G [4,11,26,46], 27-hydroxy-13-desmethyl spirolide C [47], spirolide C, spirolide D [45], 13,19-didesmethyl spirolide C [8,28], spirolide C and iso-spirolide C [28]. However, in Galicia and Portugal, until now, only 13desmeSPXC had been reported [7,33,48,49], this being the first report of a different spirolide on the Western Coast of the Iberian Peninsula.

*Mytilus galloprovincialis* was the species most affected by 13desmSPXC; in contrast, *V. corrugata*, *R. decussatus*, and the pectinid *A. opercularis* were the least affected. There are only a few comparative studies on spirolide accumulation in different bivalve species, but our results do not coincide with some of them. In Western Brittany (France), cockles (*C. edule*) had higher levels of 13desmSPXC than mussels (*M. edulis*) and clams (*Ruditapes* spp.) [50], and the same trend was found in a study covering the whole of Europe [31]. Nevertheless, both studies involved a substantially smaller number of analyses than this work; therefore, their results likely have a higher level of uncertainty. The inter-specific differences in the isomer did not follow the same trend as that of the main toxin. The isomer was only detected in mussels in three cases (out of more than 2000), and always at very low concentrations. Its highest levels were found in cockle, followed by two clams (*V. corrugata* and *P. rhomboides*), suggesting that the isomer is formed by species-dependent transformation, which is very intense in cockles, but is nonexistent or nearly nonexistent in other bivalves, such as mussels or Manila clams (*R. philippinarum*).

In general, the 13desmSPXC concentrations were positively correlated among the studied species. This suggests that the proliferation of the causing phytoplankton species encompasses the different habitats in which the studied bivalve species live. This is also in accordance with the results of spatial variability, which point to a geographically widespread distribution of the causative agent.

The spatial variation of 13desmSPXC concentrations in the bivalves was not high. In fact, the coefficient of variation of the mean concentration in the studied areas was slightly below 30%. Low spatial variability was also found along the Portuguese Coast for diverse invertebrates [49]. The grouping of all locations around the first principal component in PCA, the high percentage of the variance explained by PC1, and the low variation between species that live in different habitats also indicate the relatively low importance of spatial variation. A general slight trend in the average concentration of 13desmSPXC could be observed, with the observed levels decreasing from the southernmost areas (Atlantic Coast) to those in the northeast (Cantabrian Sea). This is also clear regarding the second principal component, for which the loadings of the different locations also follow this south-north order. The reason for this trend is not clear; however, the influence of freshwater runoff could be one of the responsible factors. In general, the Galician rivers in the Atlantic area have a higher discharge than those flowing into the Cantabrian Sea. This possible driving factor is consistent with the association of *A. ostenfeldii*, found on the coast of Maine [5] or the Baltic Sea [51], with freshwater, but is not supported by the relatively low differences found in this study between different locations inside the same Ría (with highly different impacts of freshwater), nor by the small differences among species living in different habitats.

A wide distribution of *Alexandrium ostenfeldii* and spirolides was found in the Gulf of Maine, affecting more than 150 km of the coast [5], and also in Greenland, with more than 1200 km [25]. However, in other locations, the distribution appears to be much more restricted [51,52].

In comparison with what had been previously reported, the observed seasonal pattern of 13desmSPXC was quite atypical, inasmuch as, in most cases, the maximum levels of spirolides and/or *A. ostenfeldii* had been detected in spring-summer and not in winter. On the eastern coast of Canada, for example, the maximum levels were reported in June-July [38] and May-June [19]; on Narragansett Bay (RI, USA), in April-June [53]; and on the Beagle Channel, in the austral summer [16]. In other locations, such as the Adriatic Sea [54,55] or China (Bohai Gulf) [42], a secondary maximum in autumn-winter has been reported. However, in the Bay of Biscay, the presence of *A. ostenfeldii* [56], and PSP events likely associated with it [57], were recorded only during late-autumn and winter. 

The seasonal pattern of Iso-13desmSPXC concentration was very different from that of 13desmSPXC, with maximum levels attained in spring-summer. This was also observed, even when analyzing only the periods in which the two compounds were monitored. This could be linked to the physiological conditions of the bivalves that biotransform 13desmSPXC. The possible relationship with the environmental variables has not been extensively studied, and no obvious link has been found with temperature, salinity, in vivo fluorescence, run-off, or upwelling index. Nevertheless, it seems that the start of the increase in the isomer concentration roughly coincides with the increase in the sea surface temperature [58].

Concentrations of both 13desmSPXC and its isomer decreased in the time period studied; however, similar to seasonal variation, no environmental driver has been identified.

## 4. Conclusions

13desmSPXC and one isomer have been detected in the commercial bivalves of Galicia during this 8-year study. The isomer was not found in all species, and its collision-induced dissociation (CID) MS2 and MS3 spectra were very similar to those of 13desmSPXC, suggesting that it could be a stereoisomer. The observed levels of both toxins were always low (below 50 µg kg^−1^). The mussel was the bivalve most affected by 13desmSPXC, but it was free of its isomer, whose maximum average levels were found in the cockle. The fact that 13desmSPXC content was higher in mussels than in any other commercial bivalve species studied supports its use as a sentinel species for this toxin in the area. There was a decreasing trend in 13desmSPXC concentration from south to northeast, but the temporal variation was relatively more important than the spatial variation, as the changes in concentration in the different locations were relatively synchronic. The 13desmSPXC presents a seasonal pattern, with a maximum expression in autumn-winter, but its isomer presents a quite different pattern, with a maximum expression in spring-winter, likely related to the metabolic activity of the bivalves. Both toxins showed a decreasing trend during the study period; however, no environmental factor has been associated with it.

## 5. Material and Methods

### 5.1. Chemicals, Solvents, and Reference Materials

Acetonitrile (MeCN) was obtained from Merck (Darmstadt, Germany), methanol (HPLC grade quality) from Sigma-Aldrich Chemie GmbH (Steinheim, Germany), and ultrapure water from a Milli-Q A-10 system (Millipore Iberica, Madrid, Spain).

Analytical grade ammonium hydroxide (NH_4_OH, 25%) and sodium hydroxide (NaOH > 99%) were obtained from Merck (Barcelona, Spain) and hydrochloric acid (HCL, 37%) from Panreac (Barcelona, Spain).

The 13desmSPXC; 13,19-didesmSPXC and 20-methSPXG solutions in methanol were purchased from CIFGA laboratorio S.A. (Lugo, Spain)

### 5.2. Sampling

Samples of several shellfish species were collected between January 2014 and December 2021. The mussels *Mytilus galloprovincialis* (raft-cultured and wild) were used as sentinel organisms and sampled at least weekly. Other bivalve species were sampled only when any EU-regulated toxin was detected in the mussels. As a result of this sampling strategy and of the local abundance of the different species studied, the mussel was the species most represented in the samples, followed by the cockle *Cerastoderma edule* and the carpet shell clam *Venerupis corrugata*. Other species have been analysed less frequently (the clams *Ruditapes philippinarum* and *Polititapes rhomboides*, the razor clams *Ensis siliqua* and *Ensis arcuatus*, and the pectinid *Aequipecten opercularis*). The mussel samples were obtained from two different habitats: raft-cultured and wild. Cultured mussels are grown in ropes, typically 10-m long, hanging from rafts that are located in deep areas (deeper than the rope length), and wild populations grow on rocky substrates in the intertidal zone. Most other species grow in the intertidal zone, with the exception of *P. rhomboides,* which is a subtidal species. 

Most samples were obtained from the Atlantic Coast of Galicia (14 areas, from Baiona to Cedeira), but the Cantabrian Coast of Galicia was also sampled (5 areas, from Cariño to Ribadeo) (Figure 2). Until December 2018, the isomer of 13desmSPXC was not analysed.

Samples of mussel cultures from the Galician Atlantic Coast were routinely collected, at least weekly. The production areas on the northern coast of Spain were only sampled when the harvesting of bivalve molluscs was allowed.

The 13desmSPXC was analysed in all samples, but all spirolides (Table 2) were only analysed in a subset.

### 5.3. Extraction and Sample Preparation

For representative sampling, 100–150 g of mussel soft tissues (previously rinsed with fresh water) was homogenised using a blade homogeniser. The extraction was carried out following the standard operating procedure of the EU-RL for the determination of marine lipophilic biotoxins in molluscs [59]. Aliquots of 2 g of homogenised tissues were vortexed twice with 9 mL MeOH for 60 s. After each extraction, the slurry was centrifuged at 2000 g (4 °C) for 10 min. Both supernatants were combined and the final volume adjusted to 20 mL with methanol. An aliquot was then filtered through a 0.22 µm syringe filter (PVDF 0.22 µm Millipore, Madrid, Spain), diluted with methanol (1:1 *v*:*v*), and finally analysed by LC-MS/MS.

### 5.4. LC-MS/MS Quantification

The procedure used was based on the method of Regueiro et al. [60], which was validated and optimised in INTECMAR following the standard operating procedures of the EU-RL for the determination of marine lipophilic biotoxins in molluscs by LC-MS/MS version 5 [59]. The method was accredited following the norm UNE-EN ISO/IEC 17025 (Accreditation Nº 160/LE 394) for EU-regulated marine biotoxins, but spirolides have not yet been included in the accreditation.

An Acquity UPLC coupled to a Xevo TQ-S triple quadrupole mass spectrometer through an electrospray interface (Waters, Barcelona, Spain) was used. For chromatographic separation, an Acquity BEH C18 (2.1 mm × 100 mm, 1.7 µm) column (Waters, Barcelona, Spain), maintained at 45 °C, was used at a flow rate of 400 µL min^−1^. The elution of the toxins was achieved using a binary gradient of phase A (water) and B (MeCN 90%), both with 6.7mM NH_4_OH (pH 11). The gradient started at 25% B (for 1.66 min), followed by a linear increase to 95% B at 4.3 min, and then held until minute 6.28. Subsequently, the chromatographic conditions were returned to the initial values and maintained for 2 min to equilibrate the column before the next injection. The injection volume was 5 µL.

The mass spectrometer was operated in the MRM positive ionisation mode, with the following parameters: 1 V capillary voltage, 450 °C solvation temperature, 850 L h^−1^ N_2_ flow and 150 L h^−1^ cone gas flow, and 60 V cone voltage. Two transitions were selected for monitoring each toxin (Table 2).

The 13desmSPX C was identified and quantified by comparison with a quality controlled standard (QCS) obtained from CIFGA, S.A. (Lugo, Spain), which has a purity ≥ 96%. An individual stock solution of the toxin was prepared in methanol and stored in glass vials at −20 °C. Different working standard solutions were prepared by appropriate dilution in methanol and stored in glass vials for one week. Iso-13desmSPXC was quantified by assuming the same response as 13desmSPXC in the mass spectrometer.

The response of 13desmSPXC was linear (R^2^ ≥ 0.90). Recovery ranged from 74 to 80 in mussels and cockles, and from 99 to 108 in clams. LOQ was 0.29 µg·kg^−1^ and LOD was 0.09 µg·kg^−1^.

### 5.5. LC-MS2 and LC-MS3

MS2 and MS3 fragmentation spectra were obtained using a QTRAP 6500+ triple quadrupole and linear ion trap (LIT) mass spectrometer, coupled to an Exion AD chromatographic system (SCIEX, Framingham, MA, USA) through an IonDrive Turbo V interface. The nebulizer temperature was set at 650 °C; gas 1 and 2, to 65; and the ionization voltage to +5000 V. The Enhanced Product Ion (EPI) mode was used with the following parameters: collision energy: 80, collision gas: “medium,” collision spread: 10, excitation energy 0.1, scan speed: 1000 Da s^−1^.

### 5.6. Statistical Analysis

All statistical analyses, ANOVA, Tukey HSD tests—for differences between species and habitats—and linear regression were carried out with R [61]. Principal component analysis was performed using the R packages FactoMineR [62] and factoextra [63].

## Figures and Tables

**Table 1:** Fitting of the obtained MS2 and MS3 spectra of Iso-13-desmethyl spirolide C relative to 13-desmethyl spirolide C as estimated by a Sciex Analyst library search.

Spectrum	Collision Energy	Fit	Reverse Fit
692.5 (MS2)	60 ± 10	91.1	87.5
692.5 > 444.3 (MS3)	60 ± 10	92.8	92.6
692 > 164.2 (MS3)	60 ± 10	89.7	84.1

**Table 2:** MRM transitions for the studied spirolides.

Toxin	MS/MS Transition (*m/z*)		CE (eV)
13desmSPXC and Iso-13desmSPXC	692.5>	164.3	42
		444.3	36
13,19 didesmethyl SPX C	678.5>	164	42
		430.3	36
20-methyl SPX G	706.5>	164	42
		346.3	36
Iso 13,19 didesmethyl SPX C	680>	164.1	42
		432.3	42
27 oxo 13,19 didesmethyl SPX C	692.5>	178.1	42
		444.3	42
SPX H	650.5>	164.1	40
		402.3	40
SPX I	652.5>	164.1	40
		402.3	40
SPX A	692.5>	150	50
		444.3	36
SPX G	692.5>	378.2	36
SPX B	694.4>	150	50
		444.3	36
13 desmethyl SPX D	694.4	444.3	36
		164	50
27 Hydroxy 13,19 desmethyl SPX C	694.5	180.1	40
		446.4	40
SPX C and Iso-SPX C	706.5>	164	50
		440.3	36
SPX D	708.5>	164	50
		458.3	36
27-Hydroxy-13-desmethyl SPX C	708.5	180.1	40
		460.4	40

## Data Availability

Not applicable.

## Supplementary Materials

The following are available online at https://www.mdpi.com/article/10.3390/toxins15010013/s1, Figures S1 and S2: MS3 spectra of 13desmSPXC and its isomer; Figure S3: Matrix of correlations of 13desmSPXC concentration between bivalve species; Figure S4: Correlations and regressions of 13desmSPXC concentration between bivalve species; Figure S5: 13desmSPXC concentration in wild mussels from the studied locations; Figure S6: 13desmSPXC concentration in cockle from the studied locations; Figure S7: Seasonal variation of the mean and maximum levels of 13desmSPXC and its isomer along the period of study in which the two compounds were analyzed; Table S1: number of samples analyzed for 13desmSPXC per year and month; Table S2: Number of samples analyzed for Iso-13desmSPXC per year and month; Table S3: Number of samples analyzed for 13desmSPXC and its isomer per bivalve; Table S4: ANOVA of the spatial and temporal variations.
