# Supplementary material for: Spirolides in Bivalve Mollusk of the Galician (NW Spain) Coast: Interspecific, Spatial, Temporal Variation and Presence of an Isomer of 13-Desmethyl Spirolide C
Source: Toxins (Basel). 2022 Dec 24;15(1):13. doi: 10.3390/toxins15010013 (PMC9861247; doi:10.3390/toxins15010013)
Supplement: Supplementary file 1 [file toxins-15-00013-s001.zip › toxins-2076007-supplementary.pdf]

toxins

Article

Spirolides in Bivalve Mollusk of the Galician (NW Spain) Coast: Interspecific, Spatial, Temporal Variation and Presence of an Isomer of 13-Desmethyl Spirolide C

Juan Blanco, Fabiola Arévalo, Ángeles Moroño, Jorge Correa, Araceli E. Rossignoli and Juan Pablo Lamas

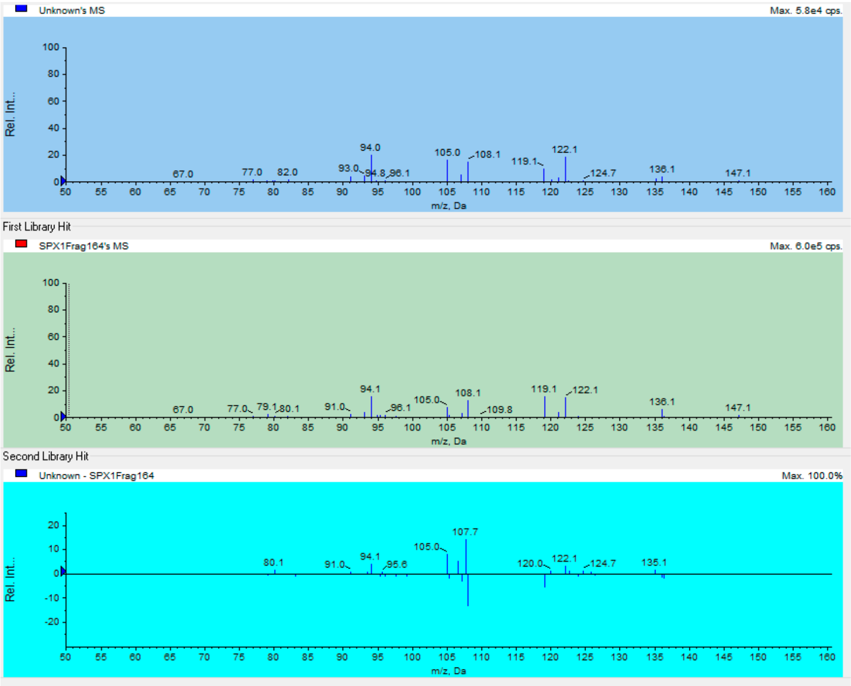

Figure S1. MS3 fragmentation spectra of the 13-desmethyl spirolide C. Fragmentation of the 164 *m/z* fragment (top panel), its isomer (middle panel) and differences between them (lower panel).

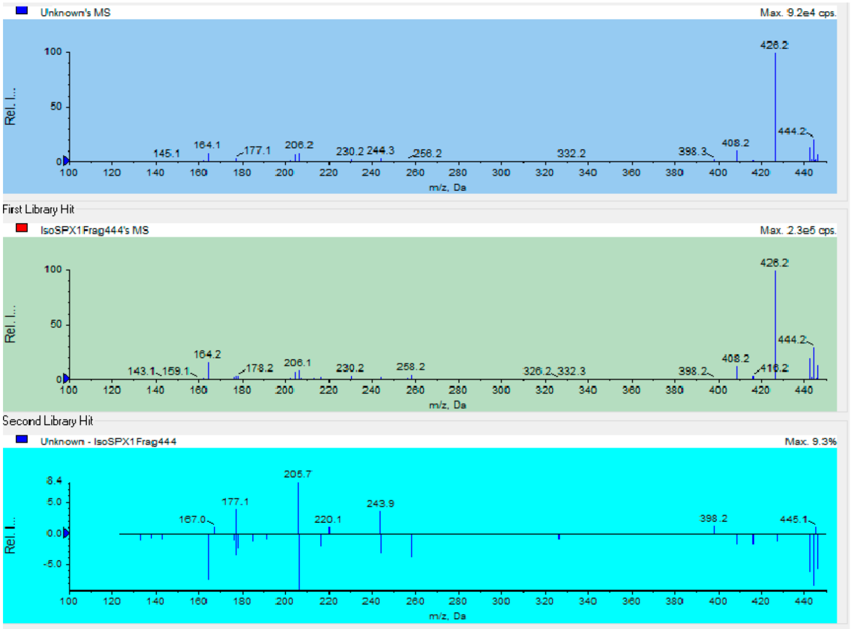

Figure S2. MS3 fragmentation spectra of the 13-desmethyl spirolide C. Fragmentation of the 444 *m/z* fragment (top panel), its isomer (middle panel) and differences between them (lower panel).

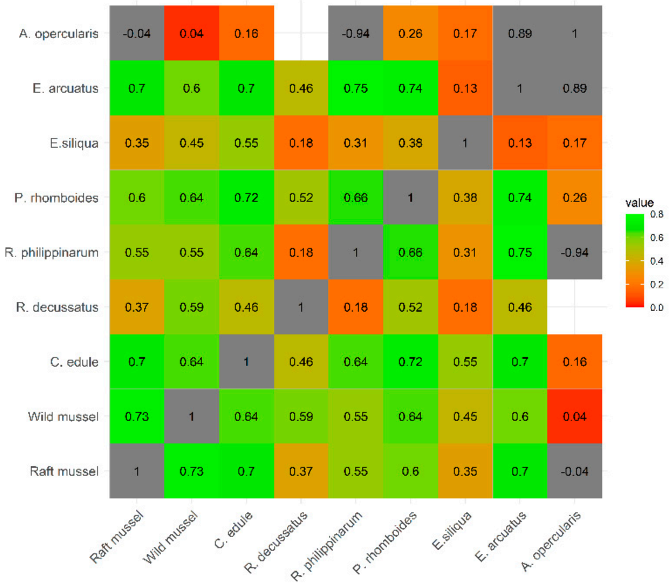

Figure S3. Matrix of correlations of 13desmSPXC concentration between bivalve species.

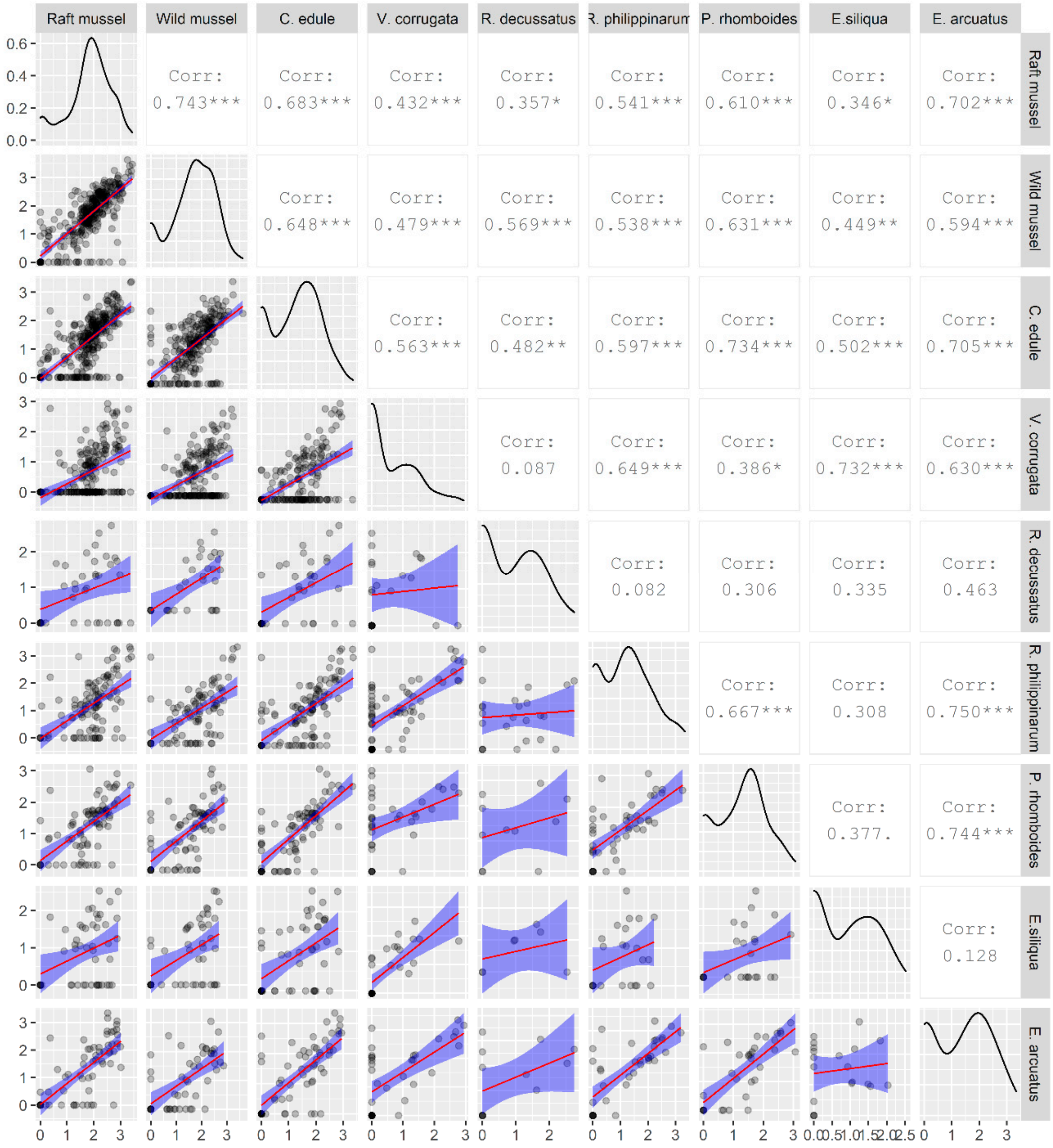

Figure S4. Correlations and regressions of 13desmSPXC concentration between bivalve species.

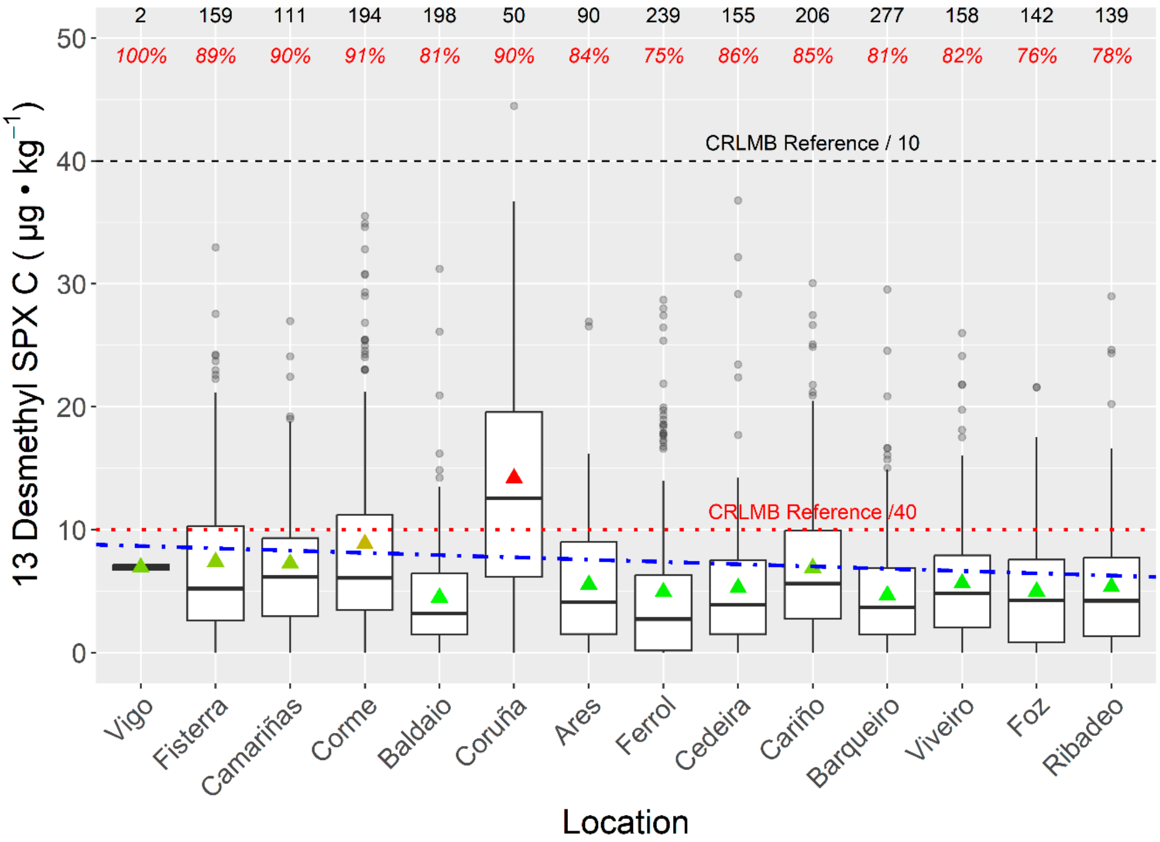

Figure S5. 13-desmethyl spirolide C concentration in wild mussels from the studied locations. Boxes and other symbols as in figure 6. Numbers at the top are the number of samples analyzed and the percentages of samples in which the compounds were detected (prevalence). The blue dashed-dotted line shows the south-northeast trend.

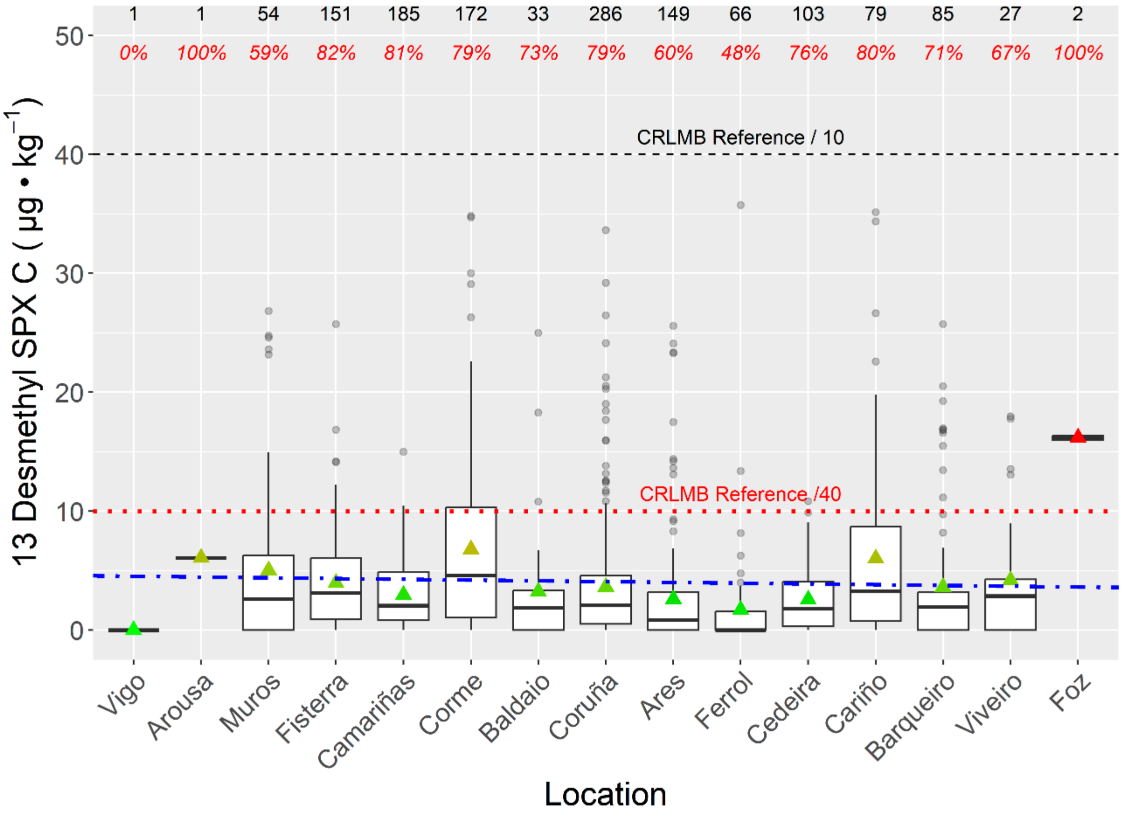

Figure S6. 13-desmethyl spirolide C concentration in cockle from the studied locations. Boxes and other symbols as in figure 6. Numbers at the top are the number of samples analyzed and the percentages of samples in which the compounds were detected (prevalence). The blue dashed-dotted line shows the south-northeast trend excluding the locations with less than three observations..

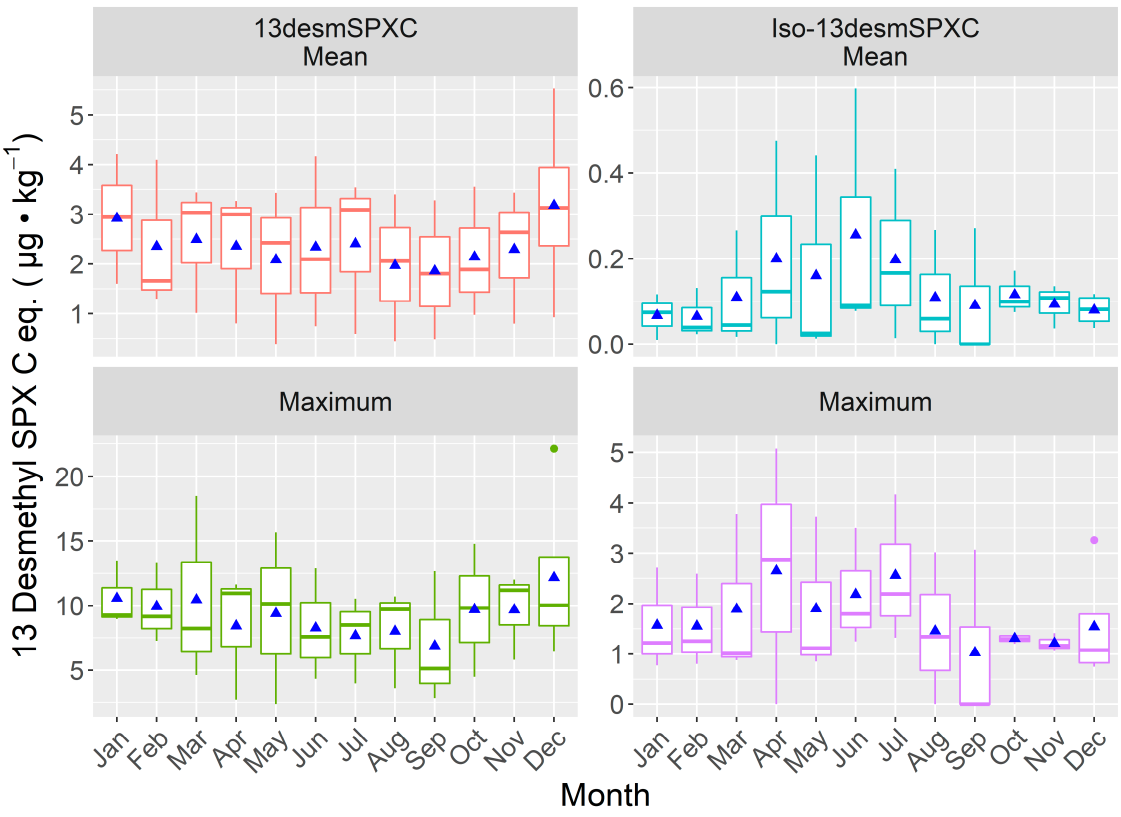

Figure S7. Seasonal variation of the mean and maximum levels of 13-desmethyl spirolide C and its isomer along the period of study in which the two compounds were analyzed. Boxes and other symbols as in figure 6.

Table S1. Number of samples of 13-desmethyl SPX C analyzed by year and month.

Year	Jan	Feb	Mar	Apr	May	Jun	Jul	Aug	Sep	Oct	Nov	Dec
2014	41	8	37	41	63	62	85	84	84	100	109	100
2015	156	146	178	117	104	161	113	151	155	127	109	114
2016	114	120	143	114	117	122	127	134	113	115	130	121
2017	131	117	141	131	134	140	160	165	155	157	131	113
2018	129	92	91	118	109	86	103	111	111	138	110	97
2019	112	98	87	82	70	90	123	94	109	112	93	90
2020	96	80	79	48	64	107	93	94	86	78	88	79
2021	78	88	117	91	74	89	102	108	82	97	93	91

Table S2. Number of samples of Iso-13-desmethyl SPX C analyzed by year and month.

Year	Jan	Feb	Mar	Apr	May	Jun	Jul	Aug	Sep	Oct	Nov	Dec
2018	--	--	--	--	--	--	--	--	--	--	--	92
2019	112	98	87	82	70	90	123	94	109	112	93	90
2020	96	80	79	48	64	107	93	94	86	78	88	79
2021	78	88	117	91	74	89	102	108	82	97	93	91

Table S3. Number of samples of 13-desmethyl SPX C and its isomer by bivalve.

Nome_Galego	13-desmSPXC	Iso-13-desmSPXC
Raft mussel	5778	2017
Wild mussel	2120	626
C. edule	1394	484
V. corrugata	301	125
R. decussatus	65	29
R. philippinarum	167	42
P. rhomboides	113	12
E.siliqua	62	11
E. arcuatus	116	8
A. opercularis	31	-

Table S4. ANOVA of the spatial and temporal variations.

```
                Df  Sum Sq Mean Sq
Location        17  14327   842.8
Week           337 106634   316.4
Location:Week 4008  68873    17.2
```
